# Topics of the Month

**Published:** 1893-11

**Authors:** 


### TOPICS OF THE MONTH.

The announcement of the College of Physicians and Surgeons of Ontario, for the academic year 1893–'94, is issued. This is the official record of the action of the Council of the Medical Profession of Ontario for the period indicated. In contains the names of officers, boards of examiners, committees, etc., of the council, together with full list of students of medicine who have passed the matriculation examination before the examiners appointed by the council; those who have passed the first, second, and third years', and the primary examinations, together with members of the College of Physicians and Surgeons, who have attained their membership by passing the final examination before the board of examiners. The minutes of the proceedings of the council are also incorporated in this volume, together with much valuable information relating to the functions and operations of this body. It is such a list as we hope the State of New York will soon publish annually for the information of the profession.

---

Brigadier-General George M. Sternberg, Surgeon-General United States Army, has inaugurated a plan for the establishment of an army medical school. General orders were issued from the war department on June 24, 1893, on this subject, and the regulations for the government of the school were announced as follows: Colonel Charles H. Alden, Assistant Surgeon-General, United States Army, president of the faculty and lecturer on the duties of medical officers; Lieutenant-Colonel William H. Forwood, Deputy Surgeon-General, United States Army, professor of military surgery; Major John S. Billings, Surgeon, United States Army, professor of military hygiene; Major Charles Smart, Surgeon United States Army, professor of military medicine and director of the chemical laboratory; Captain Walter Reed, Assistant Surgeon, United States Army, professor of clinical and sanitary microscopy and director of the pathological laboratory; Captain Julian M. Cabell, Assistant Surgeon, United States Army, assistant to the professor of military surgery and instructor in hospital corps drill.

It is further announced that the course of instruction will be for four months, and will be given annually at the Army Medical Museum in Washington City, commencing on the first day of November, 1893. It will include lectures on and practical instruction in—1. The Duties of Medical Officers in War and Peace. 2.

Military Surgery, the Care of the Wounded in Time of War, and Hospital Administration. 3. Military Hygiene. 4. Military Medicine. 5. Microscopy, Sanitary and Clinical; Pathological Histology, Bacteriology, and Urinology. 6. Hospital Corps Drill and First Aid to the Wounded. By permission of the Surgeon-General, medical officers of the army who desire to avail themselves of the course of instruction, and who are stationed in or near the City of Washington, or who have a leave of absence which enables them to attend the course, may be admitted as pupils under the same regulations as apply to recently "approved candidates for admission to the medical corps of the army." At the termination of the course of instruction, the "approved candidates for admission to the medical corps of the army" will be examined by the several professors, and their relative proficiency in each branch will be reported by the president of the faculty to the Secretary of War through the Surgeon-General of the army.

---

The Colorado Medical Library Association, at Denver, is endeavoring to build up a medical library in that city. A committee of the medical profession, consisting of Dr. J. T. Eskridge, President, Dr. Henry Sewall, Secretary, and Dr. J. C. Dana, Librarian, has addressed a circular to the several medical journals throughout the United States, as well as to a large number of the profession, inviting the presentation of books to aid in the establishment of the medical library proposed.

We commend the subject to any physician who may have volumes, either singly or in duplicate, that he feels willing to place on the shelves of the Denver library. The association will pay transportation on any such gifts, and they should be addressed "Care of the Public Library, Denver, Colorado."

---

The Philadelphia Polyclinic announces that a week will be devoted to the consideration of cataract. Lectures will be delivered, operations will be done, and clinical studies will be carried on in the wards, operating rooms and clinics of the Polyclinic, Wills Eye Hospital, and other hospitals with which the members of the teaching staff are connected. The course will be of special interest to those who are already engaged in the practice of ophthalmology, and there will be opportunity to refer to the museums and public libraries of Philadelphia in the pursuit of this or any other special study. The course begins on Monday, October 30th, and continues during the morning and afternoon of each day up to and including Saturday, November 4th. The fee for the course will be $15, and to students already taking the regular course on diseases of the eye at the Polyclinic, $5.

Information may be had on the subject by addressing Dr. A. W. Watson, Secretary Polyclinic Hospital, Lombard street, west of 13th, Philadelphia, Pa.

---

Dr. and Mrs. Charles H. Shepard, of Brooklyn, celebrated the thirtieth anniversary of the establishment of Turkish baths in this country, by a dinner given at their residence on Columbia Heights, Brooklyn, on Friday evening, October 6, 1893. The company included physicians, clergymen, merchants, lawyers, and journalists, with their wives. Dr. Shepard delivered a post-prandial address, in which he reviewed the ancient origin, the development, and the progress of the Turkish bath up to the time of its introduction into this country. He said he had been induced to launch the enterprise amid mammoth discouragements, and at first without much countenance from his brethren of the medical profession. He pointed out the advantages of the bathing system under a variety of circumstances, as a luxury, as a preventative of diseases, and as a cure for many different ailments, all of which had been amply demonstrated. Letters of regret were read from many persons from a distance, and other addresses, setting forth the beneficial experiences of the bath, were given.

This interesting occasion must have been very gratifying to Dr. and Mrs. Shepard, for the company were most cordial in compliments expressed to them for the great care and uniform courtesy which had marked their treatment of all who had betaken themselves to the influences of the Turkish baths on Columbia Heights.

---

Mr. Ernest Hart, editor of the *British Medical Journal*, who recently visited this country as the guest of the Pan-American Medical Congress, has become involved in a wordy controversy with Dr. William A. Hammond, Surgeon-General United States Army (retired).

Dr. Hammond's letters have appeared in the *New York Medical Journal*, and so have Mr. Hart's ; but in addition, Mr. Hart has seen fit to address at least one of his replies to Dr. Hammond to the entire list of medical journals in the United States. At any rate, we judge this to be the case, for we received such a communication in the form of a reproduction of a type-written letter, but signed by Mr. Hart autographically, and posted to us in a one-cent newspaper wrapper. It will thus be observed that Mr. Hart has little ethical respect for our postal laws. We notice that many of the journals throughout the land are publishing Mr. Hart's letter, though they have not published Dr. Hammond's communication which called forth Mr. Hart's answer.

We cannot understand the kind of journalistic ethics which warrants this freedom ; nor do we understand how Mr. Hart could expect to obtain so much advertising gratis. The explanation must be found, we presume, in the fact that many American medical journals have felt so highly honored with Mr. Hart's communication, that they have made haste to publish it, not thinking how rude it was to do so without having published previously Dr. Hammond's letter.

Whatever else Mr. Hart may be, or may fail to be, he is yet a very clever advertiser, which all must confess.

---

Among the novelties in the way of ambulances, is an ambulance car that is to be introduced upon the electric street car system of St. Louis. It seems that the health commissioner, Dr. George Homan, has devised a plan that the car company are giving him cordial support to perfect. It consists of an emergency hospital on wheels, with the springs so constructed as to prevent severe jars to the occupants of the car. We are looking forward to the practical trial of this system, from which we expect much good will come. Should expectations be met, it will undoubtedly be introduced in other cities, and we call the attention of the Buffalo Street Railway Company to the fact, for it should be early in the field with all useful and humane improvements like this.

---

The *Cincinnati Lancet-Clinic*, under date of October 14, 1893, speaks editorially on the subject of a state board of medical examiners, and very pertinently asks what is to be thought of a medical college that will accept of a student's fees for tuition and graduation, and then not give him such an amount and quality of instruction as to enable him to pass the ordinary examination of a state board of examiners. The *Lancet-Clinic* intimates that there are such colleges not far away from Cincinnati, and at the same time hopes that a law will be passed in Ohio that will soon regulate the matter satisfactorily.

It seems that Ohio is the only large or populous state in the Union that is not now operating under a state board of medical examiners, but we hope that it will not delay much longer in joining the procession; for if there is any state in the Union that needs the beneficent effect of a state board, it certainly is the Buckeye State.

---

The Twenty-seventh National Encampment of the Grand Army of the Republic was held at Indianapolis in the early days of September, 1893. It is highly probable that at no former encampment was its medical department organized on so comprehensive and efficient a scale as at this meeting.

The medical director, Dr. E. S. Elder, of Indianapolis, superintended the organization, and its personnel consisted of a volunteer force of 160 resident physicians and surgeons, and twenty surgeons of the G. A. R. posts in Indiana. To those were added seven contract surgeons, seven hospital stewards, four ambulance surgeons, twenty-one stewards for emergency stations, eight ambulance drivers, one medical purveyor, four bicycle couriers, and four volunteer mounted couriers—a total force of 237. The work was divided into ten divisions, and a chief surgeon was assigned to each department. On the day of the parade twenty-two emergency stations were established along the line of march. These stations rendered medical assistance to 126 cases during the day, and it is highly probable that the lives of many veterans were saved by the timely assistance then and there so promptly rendered.

There is much more of the details of this organization that we might give, had we the space at command. We have extracted this much from the comprehensive report of Dr. Elder, published in the *Indiana Medical Journal* for October, and we commend the example of the Indianapolis profession to any city that in future may aspire to the honor of entertaining the veterans.

---

The Bulletin of the Harvard Medical Alumni Association for 1893 is before us. It is No. 5 of the series, and contains a report of the third annual meeting, held in Boston, June 27, 1893. It is printed after the excellent fashion of the former bulletins, and contains, among other things, the admirable address of Dr. J. M. Da Costa, which has already been published in the *Medical News*. The bulletin can be obtained by application to the secretary, Dr. Augustus Thorndike, 101 Beacon street, Boston, Mass.

---

A PETITION has been sent to the board of supervisors, signed by twenty-four physicians of Buffalo, offering their services gratuitously as an attending staff to the Erie county almshouse, provided that the almshouse be converted into a county hospital. The idea is not new, but has been under discussion at different times, though no action has ever been taken because of serious obstacles, which have now, however, apparently been removed. The object of this move is to give the inmates of the almshouse hospital a better service, and to lift the institution beyond the pale of politics. Scarcely had the new idea been given to the public, before a move was made to invest in the chairman of the board of supervisors the power to name a committee to have full control over the proposed hospital. The most important function of this committee would be to appoint a staff of physicians, and then the hospital would be launched right in the maelstrom of politics. Of the two methods proposed, the original seems to be the more humanitarian, the physician and the patients reaping the benefits, while if the other be adopted, the politician and the politico-doctor will be the gainers. We have no wish in the matter beyond the desire that patients shall obtain the best possible service—the service they themselves prefer.

